# Current therapy in children and adolescents with von Willebrand disease

**Published:** 2014-06-25

**Authors:** I Buga-Corbu, C Arion

**Affiliations:** * "Carol Davila" University of Medicine and Pharmacy, Bucharest; ** "I. C. Fundeni" Pediatric Clinic,"Carol Davila" University of Medicine and Pharmacy, Bucharest

**Keywords:** Von Willebrand disease, prophylaxis and treatment, children and adolescents

## Abstract

Abstract

The article represents a review of recent data about the therapy of von Willebrand disease in children and adolescents (hereditary as well as acquired forms of the disease). The treatment of bleeding events in these patients, the indications in different subtypes, and the future lines of research are mentioned.

Abbreviations: Ag VWF = von Willebrand antigen, VWD = von Willebrand disease, DDAVP = arginine-vasopressin, EACA = epsilon aminocaproic acid, VWF = von Willebrand factor, VIIc F = the VIIth factor of coagulation, VIIIc F = the VIIIth factor of coagulation, IgG = immunoglobulin G, T/2 = half-time, WVF – Rco = the ristocetin cofactor.

## The treatment of von Willebrand disease The treatment of hereditary VWD The objectives of therapy and specific indications in VWD bleedings

The objective of the therapy in VWD deals with the correction of the abnormal platelets which are dependent on VWD and on the coagulation anomalies, by raising VWD and VIIIc F [6,24,25] plasmatic levels. 

 The main treatment indications are the following [12,16]: 

 • stopping the spontaneous or post-traumatic hemorrhagic episodes 

 • prevention on intraoperative/postoperative and postpartum bleedings. 

 Unlike Hemophilia A, the chronic prophylactic administration of VWF/VIII F is not necessary [**20**]. 

 The mucous-cutaneous bleedings answer to the correction of the platelet adhesion deficits by raising the levels of the VWF [**2**] multimers. 

 Intraarticular hemorrhages and in the soft tissues usually answer to the correction of circular VIIIc F levels [**4**]. 

 The specific indications, the initial doses of VWF and the duration of administration are presented in Table 1 [**2,4,5**].

**Table 1:** Specific therapeutic indications in VWD

Hemostatic situation	The objectives and duration of therapy	The VWF* initial dose and the administration frequency
Bleeding or major surgery	Realizing the levels of VIIIc F >50 iu/dL until the healing process is completed, usually for 5-10 days	50 iu/Kgc, daily (one sniff)
Minor surgery	Realizing the levels of VIIIc F >30 iu/dL until the healing process is completed, usually for 2-4 days	40 iu/Kgc, daily (one administration) or at 2 days
Minor bleeding	Realizing the levels of VIIIc F until the stopping of the bleeding, usually for 2-4 days	25 iu/Kgc daily (one administration)
Dental extraction	The VIIIc F levels of <30 ni/dL, for >12 hours	30 iu/Kgc, only one dose
Delivery/post-partum	Realizing levels of VIIIc F of >50 iu/dL, until the healing process is completed, usually of 3-5 days post-partum	50 iu/Kgc/day, starting from the date of birth

 Therapeutic methods

 DDAVP (Desmopressin) 

 The injectable substance contains 4 mcg DDAVP/mL. Maximal therapeutic answers are realized in doses of 0,1 mcg/Kgc; the whole dose is diluated in 50 mL of physiological serum and it is administrated in an endovenous perfusion for 30 minutes. 

 In normal people or in patients with type 1 VWD, raises of the levels of VWF/VIIIc F of 2-5 x basic levels are obtained in 30 minutes after the perfusion consumes [**17,20**]. 

 Subcutaneously, the dose is of 0,3 mcg/Kgc and maximum 1,5 mL of substance is administered in each place. Similar peak levels of VWF and VIIIc F are realized, just like in the case of the i.v. administration, with a latency interval of 1,5 – 2 hours. 

 Intranasal, the vasopressin doses must be raised approximately 10 times compared to the i.v. ones! DDAVP concentrated substances (ex. 1,5 mg/mL Stimate-Aventis) are needed. The whole dose is administered divided in both nostrils. The maximum serum levels are realized in approximately 1 ½ hours and, usually, they are lower than the ones obtained in the i.v. administration (similar to the ones after a 0,2 mcg/Kgc i.v. dose). 

 The half time of the raise stimulated by VWF and VIIIc F is of approximately 5-8 hours for most of the patients; the usual interval between the administration of 2 consecutive doses is of 24 hours. 

 DDAVP administration is not generally efficient in patients with type 2 and 3 VWD. In case of patients with type 1 VWD produced by the fast clearance of plasmatic VWF, just like in 2A subtype realized by an increased factor proteolysis, the therapeutic answers are of short duration [**17,20**]. 

 The individual answer to DDAVP is generally reproducible, which allows a test to establish the elective dose (**Table 2**). 

**Table 2:** The testing to establish the therapeutic answer and the DDAVP doses

A 0,3 mcg/Kgc DDAVP dose is diluted in 50 mL FS and it is administered in an endovenous perfusion for 30 minutes, while monitoring the vital signs and the side effects (flushing, headache, etc.). VIIIc F, VWF levels: RCo (or VWF:CB) and – optional – of Ag VWF must be measured at 4 hours after the start of the perfusion [**17,20**].

 VIIIc F and VWF values at 1 hour show the maximum answer; the values at 4 hours are used to establish the T/2 of the factors and the duration of the prediction of the efficient answers (for most of the patients 5-8 hours). 

 The patients with type 1 VWD, with a VWF platelet in normal ranges most often present exaggerated peak answers (up to 20 times higher than the basic values), but have a very short T2, which is adequate for the oro-dental surgery but not for major surgeries [**17,13**]. 

 There is a dissociation between the normal answer of VWF and the insufficient and short-time answer of VIIIc F, in VWD type 2N [**17,20**]. 

 The VIIIc F answer in VWD type 2A is satisfying, but of low intensity and of very short duration for VWF: RCo [**24**]. 

 The comparison between the peak values of VWF: RCo and Ag VWF can help in the differentiation of VWD from the subtypes produced by the qualitative defect of VWF, due to the VWF quantitative deficit. 

 For minor surgery and dental extractions, the peak values of VIIIc F/VWF: RCo are of 40-60 iu/dL [**4,5,17**]. 

 For the major surgeries, the necessary peak values are generally of >100 iu/dL and the levels must be maintained to >50 iu/dL by repeating the doses at 12-14 hours, also depending on the duration of the answer to the testing [**17,20**]. 

 The side effects of DDAVP administration are the following [**4,5**]: 

 • Immediate effects – transitory headache, facial flushing, light hypotension with compensatory tachycardia (in the intranasal administration in approximately 3% of the doses), rarely accompanied by nausea, vomiting, vertigo, abdominal cramps and peripheral edema. They generally answer to the reduction of the doses or of the endovenous perfusion rhythm. 

 • Tachyphylaxis – frequent side effect, which does not always affect the DDAVP haemostatic effect. The VIIIc F and VWF levels after the second administration are 30% lower than the ones obtained after the first DDAVP dose. The answers to the subsequent administrations tend to be similar to the ones after the second dose. Approximately 20% of the patients do not have sustained efficient answers. The VIIIc F and VWF: RCo levels monitoring is recommended, and, in case of an unfavorable answer, the factor concentrates administration is adopted [**20**]. 

 • Hyponatremia and hyponatremia convulsions represent side effects that are registered especially in infants and toddlers. Moreover, there is a high risk in the case of DDAVP intranasal administration concomitantly with i.v. liquids or with the excessive oral liquids contribution. The blood ionogram monitoring and the plasma osmolality are recommended together with the restriction of water ingestion, which is free in the high risk categories of patients [**6**]. 

Substitution plasma products 

 A. Cryoprecipitate 

 1 unit of cryoprecipitate is diluted in 10 mL of physiological serum and i.v. is slowly administered. 1 unit of cryoprecipitate contains approximately 100 u VIIIc F and 100 u VWF, together with 250 mg of fibrinogen (concentrations of approximately 10 times higher than the ones in the plasma). However, it is important to highlight the risk of viral infections transmission [**15**]. 

 B. VIII F/VWF concentrates obtained from plasma (Humate-P®Aventis, Alphanate®) 

 They are standardized as far as the VIIIc F and VWF content is regarded (the quantities are maintained on the lyophilized vial. The initial dosing can be made according to the 940, 41 specified VWF content). However, most of the substances are standardized, based on the VIIIc F content; the VWF: RCo values must be monitored during the therapy in order to appreciate the VWD therapeutic answer. T/2 of the plasma values is the following: 

 • approximately 13 hours for Ag VWF 

 • approximately 7 hours for VWF: RCo (because the in vivo proteolysis converts the big dimension multimers of VWF to smaller forms, with lower activity) 

 • approximately 24 hours for VIIIc F. 

 Due to these discrepancies, there is a significant risk of thrombosis in a few days after the administration; attentive monitoring is recommended together with the adjustment of the doses or the use of pure VWF concentrates. The concentrates obtained from the plasma, subjected to viral inactivation are preferable to the cryoprecipitate due to the zero chances of infectious risk. 

 The pure VIIIc F concentrates preparated from plasma or recombined are not useful for routine use in VWD, for type 3 included, because – in the absence of a concomitant administration of VWF, the plasma clearance of the factors is fast, corresponding to a T/2 of approximately 2 hours! 

 C. Pure VWF concentrates (VWF–VHP, Biotonasfasim) are available for the treatment of some special cases [**23**] – such as the one mentioned above. 

 D. Human recombined VWF 

 It is not available for the current clinical use. 

 E. other therapeutic agents 

 Substances which contain VIIa F 

 They are indicated in the treatment of VWD patients who do not answer to the VIIIc F-VWF complex administration, in cases of severe bleedings [**22**]. 

 F. Antifibrinolytic agents 

 Epsilon-aminocaproic acid (EACA) and tranexamic acid – p.o. or i.v. – are useful in the treatment of oral bleedings associated with DDAVP or VIIIc F-VWF concentrates [**17**]. 

 The side effects include nausea, diarrhea and orthostatic hypotension. 

 These substances are not indicated in the bleedings in the upper urinary tract, due to the risk of obstructing the urinary tract! Moreover, they are not indicated in patients with active tromboembolic disease! [**20**]. 

 The treatment of menstrual bleedings 

 Severe abdominal pains and even hemoperitoneum can cause the bleeding at the middle of the menstrual cycle (from the corpus luteum). This type of bleeding is controlled by oral contraceptives [**9**]. 

 Menorrhagia (risk of post hemorrhagic acute anemia!) is responsive to oral contraceptives; the answer rate to this type of medication is of 24-73%. The i.v. high-dose estrogens can raise the VIIIc F and VWF plasmatic levels. Moreover, a dose of tranexamic acid (1g at 6 hours for 5 days, or 4g/ day in an unique dose for 3-5 days) is indicated, the start of the administration must be at the moment the menstrual bleeding begins. The side effects of tranexamic acid are rare and insignificant [**10,11**]. 

 The prophylaxis and treatment of bleedings during pregnancy, birth and postpartum 

 During the pregnancy, there is a high risk of bleeding only for type 3 VWD, the substitution therapy being indicated for these types of cases [**9,10**]. 

 Normally, VIIIc F and VWF values start rising from the second trimester, reaching, at the end of the pregnancy, twice the basic level of the patient; the plasmatic levels decrease at the basic level in one week after giving birth. 

 There is a risk of peripartum bleeding in patients whose VWF and VIIIc F values are <50 iu/dL. The monitoring and VWF-VIIIc F concentrates administration in order to raise the plasmatic levels at the moment of giving birth is necessary, being of >50 ui/dL. In these conditions, the epidural anesthesia is safe [**9,11**]. 

 The uterine cavity control and the administration of the medication in order to produce efficient uterine contractions after birth are essential in order to reduce the hemorrhagic risk. 

 Postpartum, the DDAVP administration or the concentrates for the maintenance of VIIIc F plasmatic concentration is of > 50 iu/dL, for 3-4 days [**9,11**]. 

 The late postpartum bleeding (at 2-3 weeks after giving birth!) is countered by a monitorization, the administration of DDAVP or a concentrate of VWF/VIIIc F and antifibrinolytics [**10,11**]. 

 The VWD treatment with inhibitors 

 The VWF inhibitors are the IgG polyclonal antibodies, which typically inhibit the function of VIIIc F/ VWF complex but not the one of VIIIc F in pure state. They appear rarely, exclusively in patients with type 3 VWD who have suffered repeated transfusions of plasma, cryoprecipitate or a VIII F/ VWF concentrate (the frequency reported in literature being of 2,6-9,5%). 

 The patients with large deletions of the VWF gene seem to have a higher risk of appearance as an inhibitor; there is also a familial tendency [**3,4**]. 

 The VWD inhibitors diagnosis is suggested by the refractoriness to the substitution therapy, by the low T/2 of the perfused VWF and the lack of correction of the platelet functions dependant on VWF during the therapy [**2,3**]. 

 The laboratory demonstration of the presence of the inhibitors is done in the conditions in which the plasma/ serum of the patients inhibit RIPA in a normal plasma which is rich in platelets. 

 What is important to be noted is the fact that at the administration of the substitution products that contain VWF, the patients with inhibitors can present severe allergic reactions (abdominal pains, lumbar pains, hypotension, anaphylactic shock). In these patients, the use of purified VIIIc F products is indicated [**23**]. 

 The therapy of the bleedings in patients with inhibitors imposes the endovenous perfusion with high doses of VIIIc F/ (700-900iu/Kgc/day), continuously or bolus at every 4 hours, in case of major surgeries [**17**]. 

 Moreover, rh VII F activated (Novoseven®) can be used in these cases in continuous endovenous perfusion - 20mcg/Kg/h, or in bolus /10mcg/Kgc at every 4 hours)-53. 

Table 3 synthesizes the therapeutic indications in VWD subtypes [**1,24,25**]. 

**Table 3:** The recommended treatment in VWD according to the subtype

Clinical situation	1	2A	2B	2N	2M	3	Platelet type
Severe hemorrhages Major	DDAVP	VIII F+ vWF concentrates	VIII F+ vWF concentrates	VIII F+ vWF concentrates	VIII F+ vWF concentrates	VIII F+ vWF concentrates	CT
Light hemorrhages Minor surgeries	DDAVP	VIIIF+vWF concentrates (Can answer to DDAVP)	VIII F+ vWF concentrates	VIIIF+vWF concentrates (Can answer to DDAVP)	VIIIF+vWF concentrates (Can answer to DDAVP)	VIII F+ vWF concentrates	CT
Dental surgical procedures	DDAVP+ EACA	VIIIf+vWf +EACA concentrates	VIIIf+vWf +EACA concentrates	VIIIf+vWf +EACA concentrates	VIIIf+vWf +EACA concentrates	VIIIf+vWf +EACA concentrates	CT + EACA

 The treatment of acquired VWD 

 Firstly, it consists in the therapy of the causal disease. If this is not possible, according to the mechanism that leads to the inhibition/ exaggerated use of VWF, which are the following [**8,25**]: 

 • I.v. immunoglobulins (active in 1/3 of the patients with autoimmune diseases) 

 • Plasmapheresis/ extracorporeal immunoadsorption 

 • Immunosuppression medication 

 These 3 categories of therapeutic means are indicated, especially, in cases of VWD acquired through an autoimmune mechanism [**25**]. 

 • DDAVP (Needs previous testing of DDAVP answer; approximately 1/3 of the patients with acquired VWD in case of Wilms tumor answer to the treatment) 

 • Substitutive therapy with VIIIc F – VWF concentrates [**23**] 

 Generally, the patients with acquired VWD produced by hemodynamic stress or by a raised connection to the platelets/ tumor cells; do not answer to DDAVP/ IVIG concentrates of VIIIc F-VWF [**26**]. 

 The evolution and the prognosis 

 Depend on many factors, which make the adequate appreciation difficult in individual cases. In hereditary VWD (familial), the factors which influence the evolution and the prognosis are the following [**3,5**]: 

 • The correct diagnosis and setting the subtype 

 • Subtype VWD (Type 1 VWD and most of VWD2 subtypes) present light bleedings and answer to the usual therapeutic means – DDAVP and/or substitutive treatment, the evolution and prognosis being favorable. Type 2N and – especially – type 3 of VWD presents severe bleedings, the behavior being similar to the one of hemophilia A, the functional and vital prognosis being reserved. 

 • The adequate monitoring and treatment. 

 • The answer to therapy (Most of the patients with type 1 VWD answer to DDAVP, but this answer is limited by the appearance of tachyphilaxis. In the other subtypes, the answer to the substitution therapy with VIII F – VWF concentrates is generally adequate for the prophylaxis and control of the major bleedings which have appeared in other circumstances – intra- and postpartum trauma). 

 • The appearance of some complications regarding the disease (for example the hemorrhagic shock and post-hemorrhagic acute anemia, intracranial hemorrhages, etc.) or the therapy (the transmission of some viral infections, the thrombotic risk in the substitution with substances containing VIII F –VWF or rh VIIa F, fluid retention and hyponatremia convulsions in DDAVP case, etc.). 

 In the acquired VWD, the evolution and the prognosis are determined by the causal disease and the possibility of its adequate control. In many cases (the VW syndrome determined by the hemodynamic stress or by the raised connection of VWF with the platelets/ tumor cells) the answer to the usual therapeutic means – DDAVP, the substitution therapy – being inadequate [**8**]. 

Conclusions and perspectives 

 The last 30 years have been characterized by remarkable progresses in fields like the disclosure of VWF structure and also of its molecular production mechanism in the laboratory diagnosis and the hereditary VWD therapeutic algorithm, which has favorably influenced the evolution and the prognosis of the patients [**2-4,6**]. Moreover, considerable progresses have been made regarding the nosological circumscription, the pathogenesis, the diagnosis and the treatment of the acquired VWD [**7,8**]. 

 However, there are still some aspects which need specifications, representing future directions of the research in the field [**14,21**]: 

 • the development of a database regarding the responsible mutations/deletions in hereditary VWD, also including the genetic modifications which will be developed in the future; 

 • realizing some reliable and less expensive methods of screening for VWD, necessary to the population studies, and also to the present practice; 

 • the study of the hereditary VWD epidemiology in some populations, including in our country; 

 • the development and the use in current practice of the VWF substances in human recombinant; 

 • a more efficient approach of VWD as inhibitors; 

 • finding some adequate therapeutic means for acquired VWD; 

 • the development of some specialized diagnosis and treatment centers for VWD in children and adolescents; 

 the dissemination of the present knowledge regarding VWD in the community of the pediatric practitioners, and also at the level of the entire population, with the purpose of creating some behaviors favorable to the diagnosing and a more precocious therapeutic approach of hereditary VWD. 

## References

[1] Lillicrap  D (2008). The basic science, diagnosis and clinical management of von Willebrand disease.

[2] Key  N, Makris  M Practical Hemostasis and Thrombosis.

[3] Goodnight  S, Hathaway  W (2000). Disorders of hemostasis and thrombosis: a clinical guide.

[4] Federici  A, Lee  CA (2011). Von Willebrand Disease: Basic and Clinical Aspects.

[5] Hoffman  R, Furie  B (2005). Hematology: Basic Principles and Practice.

[6] Lanzkowsky  P (2008). Manual of Pediatric Hematology and Oncology, Ed. A 5-a.

[7] Dutt  T, Burns  S (2011). Application of UKHCDO 2004 guidelines in type 1 von Willebrand Disease - a single centre paediatric experience of the implications of altered or removed diagnosis. Haemophilia. May.

[8] Majumdar  G, Phillips  JK (1993). Acquired haemophilia in association with type III von Willebrand's disease: successful treatment with high purity von Willebrand's factor and recombinant factor VIIa.. Blood Coagul Fibrinolysis.

[9] Mikhail  S, Kouides  P (2010). Von Willebrand disease in the pediatric and adolescent population. J Pediatr Adolesc Gynecol.

[10] Dietrich  JE (2007). Von Willebrand's disease. J Pediatr. Adolesc. Gynecol.

[11] Collins  PW, Cumming  AM (2008). Haemophilia.

[12] Totonchi  A, Eshraghi  Y (2008). Von Willebrand disease: screening, diagnosis, and management. Aesthet Surg J.

[13] Collins  PW, Cumming  AM (2008). Type 1 von Willebrand disease: application of emerging data to clinical practice. Haemophilia.

[14] Bolton-Maggs PH, Lillicrap  D (2008). Von Willebrand disease update: diagnostic and treatment dilemmas. Haemophilia.

[15] Josephson  CD, Abshire  TC (2006). Clinical uses of plasma and plasma fractions: plasma-derived products for hemophilias A and B, and for von Willebrand disease. Best Pract Res Clin Haematol.

[16] Mannucci  PM, Franchini  M (2009). Evidence-based recommendations on the treatment of von Willebrand disease in Italy. Italian Association of Hemophilia Centers. Blood Transfus.

[17] Michiels  JJ, van Vliet  HH (2009). Managing patients with von Willebrand disease type 1, 2 and 3 with desmopressin and von Willebrand factor-factor VIII concentrate in surgical settings. Acta Haematol.

[18] Rodeghiero  F, Castaman  G (2005). Treatment of von Willebrand disease. Semin Hematol.

[19] Rodeghiero  F, Castaman  G (2009). Optimizing treatment of von Willebrand disease by using phenotypic and molecular data. Hematology Am Soc Hematol Educ Program.

[20] Michiels  JJ, van Vliet  HH (2007). Intravenous DDAVP and factor VIII-von Willebrand factor concentrate for the treatment and prophylaxis of bleedings in patients With von Willebrand disease type 1, 2 and 3.. Clin Appl Thromb Hemost.

[21] Berntorp  E, Abshire  T (2006). The von Willebrand disease prophylaxis network: exploring a treatment concept. von Willebrand Disease Prophylaxis Network Steering Committee. J Thromb Haemost.

[22] Sucker  C, Scharf  RE (2009). Use of recombinant factor VIIa in inherited and acquired von Willebrand disease. Clin Appl Thromb Hemost.

[23] Federici  AB (2007). Highly purified VWF/FVIII concentrates in the treatment and prophylaxis of von Willebrand disease: the PRO. WILL Study. Haemophilia.

[24] Nichols  WL, Hultin  MB (2008). Von Willebrand disease (VWD): evidence-based diagnosis and management guidelines, the National Heart, Lung, and Blood Institute (NHLBI) Expert Panel report (USA). Haemophilia.

[25] Batlle  J, López-Fernández  MF (2009). Von Willebrand factor/factor VIII concentrates in the treatment of von Willebrand disease. Blood Coagul Fibrinolysis.

[26] Franchini  M, Lippi  G (2007). Acquired von Willebrand syndrome: an update. Am J Hematol.

